# Thyroid Cancer Incidence in North-East Spain, 2002–2021: A Population-Based Study

**DOI:** 10.3390/cancers18193146

**Published:** 2026-09-29

**Authors:** Maria Ayala-Conte, Neus Basté, Jan Trallero, Arantza Sanvisens, Montse Puigdemont, Anna Vidal, Aina Romaguera, Wali Guan, Mercè Fernández-Balsells, Rafael Marcos-Gragera, Jordi Rubió-Casadevall, Josefina Biarnés

**Affiliations:** 1 Department of Endocrinology and Nutrition, Hospital Universitari de Girona Dr. Josep Trueta, 17007 Girona, Spain; mayala.girona.ics@gencat.cat (M.A.-C.); mercefernandez.girona.ics@gencat.cat (M.F.-B.); 2 Medical Oncology Department, Hospital Clínic de Barcelona, 08036 Barcelona, Spain; neusbaste@gmail.com; 3 Translational Genomics and Targeted Therapies in Solid Tumours Group, Institut d’Investigacions Biomèdiques August Pi i Sunyer (IDIBAPS), 08036 Barcelona, Spain; 4 Facultat de Medicina i Ciències de la Salut, Universitat de Barcelona (UB), 08036 Barcelona, Spain; 5 Integrated Cancer Registries Service (SIRC), Catalan Institute of Oncology, 17007 Girona, Spain; jtrallero@iconcologia.net (J.T.); asanvisens@iconcologia.net (A.S.); mpuigdemont@iconcologia.net (M.P.); marianna.vidal@iconcologia.net (A.V.); aina.romaguera@udg.edu (A.R.); rmarcos@iconcologia.net (R.M.-G.); 6 Research Group on Descriptive Epidemiology, Genetics and Cancer Prevention, Girona Biomedical Research Institute Dr. Josep Trueta (IDIBGI-CERCA), 17190 Salt, Spain; jrubio@iconcologia.net; 7 Research Group on Statistics, Econometrics and Health (GRECS), University of Girona, 17003 Girona, Spain; 8 Josep Carreras Leukaemia Research Institute, 17004 Girona, Spain; 9 Department of Computer Science, Applied Mathematics and Statistics, University of Girona, 17004 Girona, Spain; 10 Medical Science Department, Faculty of Medicine, University of Girona, 17004 Girona, Spain; waliguan@bellvitgehospital.cat; 11 Nutrition, Eumetabolism and Health Group, Institut d’Investigació Biomèdica de Girona (IDIBGI-CERCA), 17190 Girona, Spain; 12 CIBER Fisiopatología de la Obesidad y Nutrición, Instituto de Salud Carlos III, 28029 Madrid, Spain; 13 Medical Oncology Departament, Catalan Institute of Oncology, Hospital Universitari Josep Trueta, 17007 Girona, Spain

**Keywords:** thyroid cancer, epidemiology, incidence, cancer registry

## Abstract

**Background/Objectives:** The incidence of thyroid cancer (TC) has increased worldwide over recent decades, particularly for well-differentiated histological subtypes, a trend largely attributed to overdiagnosis. Given potential geographical differences, this study analyzed TC incidence and temporal trends in the province of Girona, Spain, between 2002 and 2021. **Methods:** We conducted a cross-sectional study using data from the Girona Cancer Registry. Crude incidence rates (CRs), age-standardized incidence rates using the 2013 European (ASRe) and the 1960 World (ASRw) standard populations were calculated per 100,000 person-years. Temporal trends were assessed via the annual percentage change (APC) using generalized linear models. **Results:** A total of 998 incident TC cases were identified. Most patients were women (78.0%), with a median age at diagnosis of 48 years. Papillary TC was the predominant histological subtype (81.7%), and 70.6% of cases were diagnosed at Stage I. The overall ASRe was 6.8 and ASRw was 5.5, with incidence rates 3–4 times higher in women than in men. Overall, TC incidence increased significantly (APC: 2.7%), driven by increases in the 35–44 and ≥55 age groups, papillary histology, and early-stage disease (Stages I–II). **Conclusions:** The rise in TC incidence in Girona over the last two decades has been largely driven by Stage I papillary tumors, in both sexes, supporting a potentially substantial contribution of overdiagnosis. However, the concurrent increase in Stage II disease warrants continued surveillance, as it may reflect additional risk factors.

## Simple Summary

Thyroid cancer has become one of the most rapidly growing cancer diagnoses worldwide over recent decades, largely attributed to the increased detection of small, clinically insignificant tumors through improved imaging technologies—a phenomenon known as overdiagnosis. However, this rise cannot be explained by detection alone. This study examines how the incidence of thyroid cancer has changed over a 20-year period (2002–2021) in the province of Girona, Spain, using data from a population-based cancer registry. The findings confirmed a steady increase in incidence, predominantly affecting women and driven by early-stage papillary tumors, which may be consistent with overdiagnosis. Notably, a concerning rise in intermediate-stage cases and a narrowing gap between sexes in older age groups suggest that additional risk factors beyond improved detection may be at play. These results highlight the need for more tailored clinical approaches and continued epidemiological surveillance in this population.

## 1. Introduction

Thyroid cancer (TC) is the most common type of endocrine cancer. In 2022, an estimated 821,214 new cases were diagnosed worldwide, ranking TC as the seventh most frequently diagnosed cancer. However, TC accounted for only 47,507 cancer-related deaths in 2022, placing it twenty-fourth among all cancers in terms of mortality [1].

Over the past few decades, the incidence rates of TC have increased markedly worldwide, particularly among women. In high-income countries, for instance, incidence rates rose from approximately 10 cases per 100,000 women-years in the early 1980s to 60 cases per 100,000 women-years in the early 2010s. Numerous studies have examined temporal trends in TC incidence worldwide. Several countries, including South Korea, the United States, Canada, and Israel, as well as European countries, such as France, Italy, Austria, and Ireland, have revealed an increasing trend until the early 2010s, followed by a decline in some of them [2]. Meanwhile, previous research in Spain has shown a significant increase in the incidence of TC from 1978 to 2010, primarily due to the diagnosis of early-stage carcinomas [3]. 

This increase has largely been attributed to overdiagnosis, which is defined as the identification of a condition that would not have caused symptoms or death, primarily driven by the widespread accessibility of neck ultrasonography and fluorodeoxyglucose-positron emission tomography as an oncological screening technique [4]. This is further supported by the rapid yet geographically heterogeneous rise in incidence rates observed across different regions [5]. Despite the increase in incidence rates, mortality rates have remained stable or declined in many countries [6].

This rise in incidence has primarily been associated with the papillary subtype [7]. In addition, autopsy studies conducted in individuals of different ages and sexes who died from causes unrelated to TC have reported a prevalence of subclinical papillary TC of 12.9% (95% confidence intervals (CIs): 7.8, 16.8) when the entire thyroid gland was examined histologically [8].

Understanding incidence trends in TC can provide valuable insights into its evolving patterns and contribute to the understanding of its epidemiology. Given this geographical heterogeneity, local population-based data are needed to accurately describe the burden of TC in our region and to inform health planning. Therefore, the present study aims to analyze the incidence and incidence trends of TC in the province of Girona, a region in north-east Spain, during the period 2002–2021. The analysis will focus on differences according to age at diagnosis, sex, histological type, and stage at diagnosis. 

## 2. Materials and Methods

### 2.1. Study Population

This population-based study included all malignant incident cases of TC obtained from the Girona Cancer Registry (GCR) between 2002 and 2021. The GCR is part of the Spanish Network of Cancer Registries (REDECAN), the European Network of Cancer Registries (ENCR) and the International Association for Cancer Registries (IACR). Cancer registration procedures and coding rules were applied according to the standards of the International Agency for Research on Cancer (IARC) and ENCR recommendations. The GCR covers the population of the Girona province, located in north-east Spain, which had 784,941 inhabitants in 2021 [9].

All invasive primary malignant tumors located in the thyroid gland diagnosed among residents of the province of Girona during the study period were included. Hematological malignancies and sarcomas were excluded. For each case, information on sociodemographic variables (sex and date of birth) and tumor characteristics (date of diagnosis, age at diagnosis, method of diagnosis, tumor topography, morphology, and stage at diagnosis) were available. 

Tumor morphology codes according to the International Classification of Diseases for Oncology, 3rd Edition (ICD-O-3) were classified into five types as (1) papillary (codes: 8050, 8260, 8340–8344, 8350), (2) follicular including oncocytic (codes: 8290, 8330, 8332, 8335, 8339), (3) medullary (codes: 8345, 8510–8513), (4) anaplastic and poorly differentiated (codes: 8020, 8021, 8337), (5) others (codes: 8000, 8010, 8430, 8346, 8347, 8200). Age at diagnosis was grouped into five groups: <35, 35–44, 45–54, 55–64, and >64 years. Stage at diagnosis was categorized into five stages according to the American Joint Committee on Cancer (AJCC), I, II, III, IV, and unstaged, according to the edition in effect at the time of diagnosis [10,11]. During the study period (2002–2021), four AJCC TNM editions were applied: the 5th edition (2002), the 6th edition (2003–2009), the 7th edition (2010–2017), and the 8th edition (2018–2021). 

### 2.2. Ethics Statement

This study was conducted in accordance with the principles of the Declaration of Helsinki. It was reviewed and approved by the Institutional Review Board of the Hospital Universitari de Girona Dr. Josep Trueta (protocol code: GEDGPC-TIR-2022-01, approval number: 2022.141). Informed consent was not required as the study falls under the legal framework for public health research and surveillance. Specifically, the processing of data was carried out in accordance with Spanish General Health Laws (14/1986 and 33/2011), which governs the Pla Estadístic de Catalunya (extended period 2017–2020) and recognizes the GCR as an official statistical file. Furthermore, data confidentiality and privacy were strictly maintained according to the General Data Protection Regulation (EU 2016/679) and the Spanish Organic Law 3/2018 on the Protection of Personal Data and Guarantee of Digital Rights.

### 2.3. Statistical Analyses

Descriptive analyses were performed using absolute and relative frequencies expressed as a percentage for categorical variables and medians and interquartile range (IQR) for numerical data. Statistical comparisons by sex were performed using a *t*-test for numerical data and the chi-squared test and Fisher exact test for categorical data. The latter was applied only when expected counts were below 5.

Incidence rates were estimated as crude rates (CRs) and age-standardized rates (ASRs), expressed per 100,000 person-years (p-y). For the latter, direct standardization of quinquennial age-bands employing the 2013 European standard population (ASRe) and the 1960 Segi world standard population (ASRw) were used [12,13]. In addition, sex ratio (SR) was estimated as the ratio between the ASRe in women with respect to the ASRe in men.

Incidence trends were modeled through generalized linear models (GLMs) with a Poisson distribution adjusted by age and year of diagnosis. For each model, overdispersion, zero-inflation, and goodness-of-fit test for Poisson assumption were assessed. Additionally, segmented models were used to assess point changes in the trend based on the Akaike Information Criterion (AIC). From the final selected model, the annual percentage change (APC) was estimated. A sensitivity analysis was performed comparing the APCs estimated from three different models fitted with data from 2002 to 2021, 2002 to 2021 (excluding 2020), and 2002 to 2019 to assess the possible effect of the COVID-19 pandemic into the incidence trends.

The population at risk in the province of Girona during 2002–2021 was obtained from the Spanish National Institute of Statistics (Supplementary Table S1). Statistical significance was assessed at the 95.0% confidence level. All the statistical analyses were performed using R software, version 4.5.0.

## 3. Results

From 2002 to 2021, a total of 998 TC cases were diagnosed in the Girona Province. Women accounted for 78.0% of all cases (N = 778). The median age at diagnosis was 48 years [IQR: 37–60], with women being diagnosed at a younger age than men (median 46 years [IQR: 36–59] vs. 52 years [IQR: 41–63], respectively). In the overall study population, nearly half of all TC cases were diagnosed between 35 and 54 years of age. Nevertheless, women were diagnosed at earlier ages, predominantly at <55 years, in comparison with men, who were mostly diagnosed from 45 years onward (Table 1).

As seen in Table 1, a confirmed histological diagnosis was available for 99.6% of cases (N = 994). An evaluation of the histological subtypes of TC revealed that papillary TC was the most common, accounting for 81.7% of cases. Follicular TC represented 9.6%, medullary TC 5.0%, and anaplastic or poorly differentiated TC 2.6% of cases. Notable sex differences were observed in the distribution of histological subtypes: differentiated histology was more frequent in women, whereas medullary and undifferentiated histologies were more frequent in men. Regarding stage at diagnosis, 70.6% of cases (n = 704) were diagnosed at Stage I. Sex-specific differences were also evident. Stage I predominated among women, whereas men presented a higher proportion of more advanced stages at diagnosis.

### 3.1. Incidence Rates

The overall CR of TC was 7.0 per 100,000 p-y [95% CI: 6.5, 7.4] and was substantially higher in women than in men. This sex disparity persisted after age standardization using both the ASRw and the ASRe. The SR confirmed a clear female predominance (SR = 3.4 [95% CI: 2.9, 3.9]) (Table 2).

Based on the data presented in Table 2, across all age groups, incidence rates were consistently higher in women than in men, although the magnitude of this difference decreased progressively with increasing age. The greatest sex disparity was observed among individuals younger than 35 years, with a highest predominance of women compared to men (SR = 5.9 [95% CI: 3.9, 8.8]). Among older adults, this difference between sexes further diminished, reaching an SR = 2.4 [95% CI: 1.7, 3.4] in those aged >64 years.

A pronounced female predominance was observed for papillary (SR = 3.9 [95% CI: 3.3, 4.7]) and follicular TC (SR = 4.2 [95% CI: 2.5, 7.1]). In contrast, no meaningful sex differences were observed for medullary, anaplastic and poorly differentiated TC, as summarized in Table 2.

Women exhibited higher incidence rates than men across all stages at diagnosis, with the magnitude of the sex difference decreasing with advancing stages. A marked female predominance was observed for Stage I (SR = 4.8 [95% CI: 4.0, 5.9]) and Stage II (SR = 3.0 [95% CI: 1.8, 5.0]), whereas Stage IV showed no sex difference (SR = 1.2 [95% CI: 0.8, 1.7]) (Table 2).

### 3.2. Incidence Trends

The incidence trends of TC are detailed in Figure 1. Overall incidence increased significantly over the study period, with an APC of 2.7% [95% CI: 1.6, 3.8]. Increasing trends were observed in both sexes, with a seemingly higher APC in men (APC = 3.1% [95% CI: 0.7, 5.5]) compared with women (APC = 2.4% [95% CI: 1.2, 3.7]). The results of the overdispersion, zero-inflation, and goodness of fit for Poisson assumption are shown in Supplementary Table S2. In addition, across all Poisson GLMs, no point changes in the incidence trend were detected. The sensitivity analysis revealed no differences in the APCs for the 2002–2021, 2002–2021 (excluding 2020), and 2002–2019 periods.

Age-specific analyses showed the steepest increases among individuals older than 54 years, particularly in those aged >64 years (APC = 4.9% [95% CI: 2.3, 7.5]). A non-negligible rise in APC was also observed in the 35–44-year group (APC = 2.5% [95% CI: 0.1, 4.8]) (Figure 1).

The only histological subtype responsible for the upward trend of the APC amongst all cases is papillary TC (APC = 2.9% [95% CI: 1.7, 4.1]). An increasing but not significant trend was observed for anaplastic and poorly differentiated carcinoma, with an APC of 6.7% [95% CI: −0.4, 13.8]). With respect to stage at diagnosis, remarkable increases were observed only for Stage I (APC = 3.9% [95% CI: 2.6, 5.3]) and Stage II (APC = 5.2% [95% CI: 1.1–9.3]), as reported in Figure 1.

## 4. Discussion

This population-based study, including 998 incident cases of TC diagnosed in Girona Province between 2002 and 2021, revealed a significant and sustained increase in overall TC incidence, with an APC of 2.7%. This growth was observed in both sexes and was predominantly driven by papillary TC, particularly Stage I and Stage II, and mainly affected patients aged between 35 and 44 and older than 54 years old.

These patterns could be suggestive of overdiagnosis, whereby indolent tumors that are unlikely to cause symptoms or affect survival are increasingly detected. This phenomenon is widely attributed to the widespread use of neck ultrasonography or the growing availability of other diagnostic tools such as computed tomography and positron emission tomography, which are frequently performed for non-thyroid indications in clinical and oncological settings [4]. Globally, TC incidence has risen substantially over recent decades. Between 1990 and 2013, the global ASR of TC increased by approximately 20% [14]. This upward trend was primarily attributed to overdiagnosis. A prominent example is South Korea, where the implementation of a national screening program led to an APC of 24.4% in TC incidence between 1999 and 2010, predominantly driven by the increased detection of papillary TC, without any parallel reduction in mortality [15,16]. 

Weaker trends have been noted across Europe and North America. In France, data from eight cancer registries showed an increase of 6.2% per year in women and 8.1% per year in men in TC incidence between 1978 and 1997 [17]. In the Italian region of Venezia, the ASR for TC increased by 33.1% in women and 44.2% in men between 2002 and 2013 [18]. Denmark reported steady APCs (1.7% in men, 1.8% in women) from 1943 to 2008 [19]. In the United States, papillary TC incidence increased sharply between 1993 and 2012 (APC 6.26%), while follicular TC and medullary TC showed more modest rises (1.57% and 1.87%, respectively) [20].

In Spain, studies conducted in Granada (1985–2013) and Navarre (1986–2010) reported APCs slightly higher than those observed in our study. APCs increased in both sexes, predominantly driven by papillary TC (Granada: 5.4% in men, 4.7% in women; Navarre: 4.7% in men, 2.5% in women) [21,22]. This phenomenon underscores the issue of overdiagnosis although our findings indicate a more moderate rise in incidence, which may reflect differences in diagnostic practices and healthcare systems. In high-income countries, private healthcare services and liberal use of neck ultrasonography have contributed significantly to overdiagnosis. Consequently, regional disparities in TC incidence likely reflect differences in medical practice patterns, as well as urban–rural disparities [23]. Therefore, overdiagnosis may result in overtreatment, psychological distress, reduced quality of life, and an increased financial burden on the healthcare system without improved survival [24,25]. Similar trends have been observed in other malignancies with established screening programs, such as breast and prostate cancer [26,27].

Traditionally, the rise in TC has been attributed to the overdiagnosis of small incidentally detected tumors, so there have been changes in the main clinical guidelines, such as the American Thyroid Association (ATA) guidelines in 2015 [28]. Acknowledging the potential harms of overdiagnosis and overtreatment, the new guidelines now advocate for less aggressive diagnostic and therapeutic approaches [29].

In parallel with these shifts in clinical management, important modifications were also introduced in the staging system to better align risk stratification with disease-specific outcomes. The main changes focused on risk stratification were age cutoff (raised from 45 to 55 years), microscopic extrathyroidal extension removal (from T3 definition), and N1 disease status (patients are no longer upstaged to Stage III). This led to a large proportion of patients being significantly downstaged, particularly those aged 45–54 years at diagnosis, with the application of the 8th edition of the AJCC TNM staging system, implemented solely to the final four years of the study period (2018–2021) [11].

While shifts toward conservative management may have mitigated overdiagnosis in some regions, they have not influenced the incidence of more advanced or aggressive tumors. This observation suggests that both improved detection methods and a possible true increase in disease incidence may be contributing to global trends [30].

Likewise, the rising trend observed in Stage II in our study—which, from 2018 onward, includes locally advanced tumors and/or tumors with lymph node involvement—should be interpreted in the context of the changes introduced by the 8th edition of the AJCC staging system, which represents a plausible explanation for the observed increase in Stage II cases. Beyond this staging reclassification, other potential contributing factors may also warrant consideration, including environmental exposure to ionizing radiation at an early age, such as that resulting from radiological examinations or nuclear accidents, as well as environmental pollution, particularly exposure to heavy metals. Other potentially modifiable factors, including variations in dietary iodine intake and high body mass index, may also contribute to the observed trends [31,32,33,34]. Regional disparities in TC burden are likely influenced by a complex interplay of environmental, biological, and healthcare-related factors, as well as lifestyle changes and socioeconomic inequalities in healthcare access. Worldwide TC studies have underscored the importance of local sociodemographic and healthcare system characteristics in shaping TC incidence trends and outcomes [2].

TC shows sexual dimorphism, with a three- to five-fold higher incidence in women [35]. This gap, evident in our cohort based on SR, peaks during the reproductive years and narrows after menopause, suggesting estrogens as drivers of thyroid carcinogenesis [36,37]. However, sex hormones should not be regarded as the primary drivers of these differences, but rather as potential contributors, alongside other biological, demographic, and epidemiological factors. Estrogens act genomically, through nuclear estrogen receptor α (ERα) and ERβ regulating cyclin D1 (CCND1) transcription, and non-genomically, through membrane-bound receptors such as G protein-coupled estrogen receptor 1 (GPER1) [38]. ERα promotes proliferation, survival, and aggressive tumor behavior, whereas ERβ generally acts as a tumor suppressor, inducing differentiation and apoptosis [39,40]; an increased ERα/ERβ ratio drives carcinogenesis and predicts aggressiveness and poorer prognosis in papillary TC [41,42]. *BRAF V600E* acts synergistically, enhancing estrogen-induced metastatic potential by regulating ER expression [43]. Estrogen-activated GPER1 signals via PI3K/AKT/mTOR and RAS/RAF/MAPK promote survival, migration, and epithelial-to-mesenchymal transition [44]. Papillary TC risk appears to increase with prolonged high endogenous estrogen exposure, multiparity, and recent pregnancy, while breastfeeding and oral contraceptive use may confer a protective effect [44,45,46,47]. A higher risk of TC during pregnancy is likely due to elevated estrogens levels, iodine deficiency, and the TSH receptor-stimulating effects of hCG [46,48]. ER-positive breast cancer is a significant risk factor for the development of TC; therefore regular thyroid screening should be recommended for this population to mitigate the increased risk of secondary malignancy driven by genetic and hormonal factors [49].

Environmental exposure to endocrine-disrupting chemicals (EDCs) represents a growing public health concern related to TC incidence. These chemicals, such as phthalates, bisphenols, heavy metals (cadmium), per-/poly-fluoroalkyl substances (PFAS) and polychlorinated biphenyls (PCBs), can interfere with the endocrine axis and mimic or block the effects of natural estrogen, increasing the risk of TC [50,51,52].

Although papillary TC is more frequent in females, the gap in advanced stages and aggressive histologies becomes closer between sexes. These findings may be consistent with prior studies reporting higher mortality and worse prognoses in male patients [53,54,55,56]. 

A notable limitation of this study is the use of different editions of the AJCC TNM staging system over the study period (2002–2021), which introduces heterogeneity in stage classification. Four editions were applied: the 5th (2002), 6th (2003–2009), 7th (2010–2017), and 8th (2018–2021). Changes from the 5th until the 7th edition had minimal impact on overall stage distribution; however, the transition to the 8th edition resulted in substantial downstaging. Furthermore, the low number of cases in Stages II, III, and IV might contribute to a low statistical power affecting stage-specific trends in the study. Thus, it is imperative to exercise caution when interpreting stage-based analyses due to variations in staging criteria across different editions. Such disparities may compromise the comparability of these analyses over time.

Furthermore, information on tumor size and the reason for diagnosis were unavailable, preventing differentiation between clinically detected and incidentally discovered tumors. Consequently, the predominance of Stage I disease should not be considered a direct measure of overdiagnosis and the ability to quantify the extent of overdiagnosis is limited. In addition, the descriptive nature of the study precludes causal inference regarding environmental or lifestyle or risk factors.

Despite these limitations, this study represents the third population-based analysis of TC incidence in Spain, following those conducted in Navarre and Granada [21,22]. It is the first and only study to include the period after publication of the 2015 American Thyroid Association guidelines [28], which marked a significant shift toward more conservative management strategies. This inclusion allows the evaluation of recent trends in diagnosis and treatment within the context of updated clinical practice.

A key strength of this study lies in the comprehensive stratified analysis by sex, age, stage, and histological subtype, providing a detailed and nuanced picture of TC epidemiology in the region of Girona. Furthermore, it is worth noting that incidence trends based on tumor staging are included, which is not common in Spanish registry studies. This study shows demographic and clinical differences in tumor presentation, offering valuable insights into disease behavior and its variation across population subgroups. 

## 5. Conclusions

Over the last two decades, papillary TC diagnosed at Stages I and II has been the main contributor to the increasing incidence of TC in Girona province in both sexes. Consistent with findings from other studies, these trends may suggest that overdiagnosis accounts for a substantial proportion of the observed increase. Nevertheless, the increase observed in Stage II cases (APC: 5.2%) may warrant further monitoring to better understand the factors that could be contributing to changes in the disease burden beyond improved detection.

Furthermore, the female predominance was most pronounced in younger age groups, in differentiated histological subtypes, and in early-stage disease. Further research is warranted to clarify the role of sex in TC incidence and to determine to what extent this predominance reflects biological susceptibility rather than differential diagnostic scrutiny. These findings underscore the relevance of sex-specific, patient-centered approaches to TC management and reinforce the need to balance early detection against unnecessary diagnosis and treatment.

## Figures and Tables

**Figure 1:**
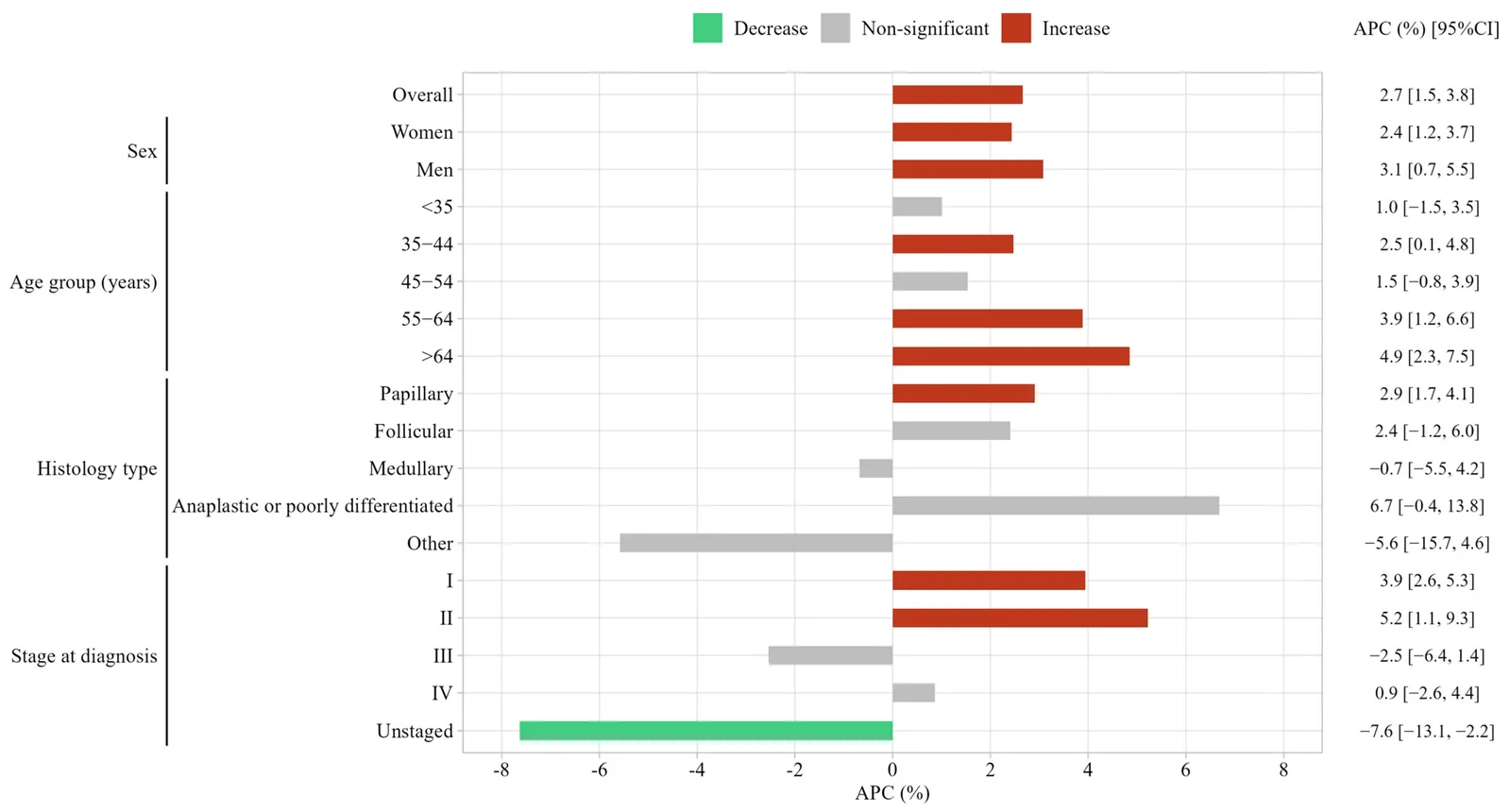
Incidence trends of thyroid cancer by age group, histology and stage, 2002-2021, Girona, Spain. Statistical significance of the APC was determined by whether the 95% CI excluded 0.

**Table 1:** Sociodemographic and tumor characteristics of overall thyroid cancer cases by sex, 2002–2021, Girona, Spain.

Characteristics	Total N = 998 n (%)	Sex		
		Women N = 778 n (%)	Men N = 220 n (%)	*p*-Value
Age at diagnosis (years)				0.002 ^1^
Mean ± SD	48.8 ± 15.7	48.0 ± 15.5	51.8 ± 15.9	
Median [IQR]	48.0 [37.0–60.0]	46.0 [36.0–59.0]	52.5 [41.0–63.0]	
<35	191 (19.1)	163 (21.0)	28 (12.7)	
35–44	232 (23.2)	187 (24.0)	45 (20.5)	
45–54	218 (21.8)	169 (21.7)	49 (22.3)	
55–64	174 (17.4)	124 (15.9)	50 (22.7)	
>64	183 (18.3)	135 (17.4)	48 (21.8)	
Method of diagnosis				-
Death certificate only	1 (0.1)	1 (0.1)	0 (0.0)	
Not microscopically verified	3 (0.3)	1 (0.1)	2 (0.9)	
Microscopically verified	994 (99.6)	776 (99.8)	218 (99.1)	
Period of diagnosis (years)				0.473 ^2^
2002–2006	179 (17.9)	140 (18.0)	39 (17.7)	
2007–2011	213 (21.3)	171 (22.0)	42 (19.1)	
2012–2016	298 (29.9)	236 (30.3)	62 (28.2)	
2017–2021	308 (30.9)	231 (29.7)	77 (35.0)	
Histology type				<0.001 ^3^
Papillary	815 (81.7)	653 (83.9)	162 (73.6)	
Follicular	96 (9.6)	78 (10.0)	18 (8.2)	
Medullary	50 (5.0)	29 (3.7)	21 (9.5)	
Anaplastic or poorly differentiated	26 (2.6)	13 (1.7)	13 (5.9)	
Other	11 (1.1)	5 (0.6)	6 (2.7)	
Stage at diagnosis				<0.001 ^2^
I	704 (70.6)	583 (74.8)	121 (55.0)	
II	77 (7.7)	58 (7.5)	19 (8.6)	
III	77 (7.7)	56 (7.2)	21 (9.5)	
IV	98 (9.8)	54 (6.9)	44 (20.0)	
Unstaged	42 (4.2)	27 (3.5)	15 (6.8)	

IQR: Interquartile range; SD: Standard deviation; ^1^ *t*-test for equal variances; ^2^ Chi-squared test; ^3^ Fisher exact test.

**Table 2:** Crude rates, age-specific rates and sex ratio of incidence of thyroid cancer according to sociodemographic and tumor characteristics, 2002–2021, Girona, Spain.

Characteristics		Sex	CR [95%CI] *	ASRw [95%CI] *	ASRe [95%CI] *	SR [95%CI]
Total		Both	7.0 [6.5, 7.4]	5.5 [5.1, 5.8]	6.8 [6.4, 7.3]	3.4 [2.9, 3.9]
		Women	10.9 [10.2, 11.7]	8.8 [8.2, 9.5]	10.6 [9.9, 11.4]	-
		Men	3.1 [2.7, 3.5]	2.3 [2.0, 2.6]	3.1 [2.7, 3.6]	-
Age group (years)	<35	Both	3.3 [2.8, 3.7]	1.6 [1.4, 1.9]	1.2 [1.0, 1.3]	5.9 [3.9, 8.8]
		Women	5.7 [4.9, 6.7]	2.8 [2.4, 3.3]	2.0 [1.7, 2.4]	-
		Men	0.9 [0.6, 1.3]	0.5 [0.3, 0.7]	0.3 [0.2, 0.5]	-
	35–44	Both	9.9 [8.7, 11.2]	1.2 [1.0, 1.4]	1.3 [1.2, 1.5]	4.5 [3.2, 6.2]
		Women	16.5 [14.3, 19.1]	2.0 [1.7, 2.3]	2.2 [1.9, 2.6]	-
		Men	3.7 [2.8, 5.0]	0.4 [0.3, 0.6]	0.5 [0.4, 0.7]	-
	45–54	Both	10.6 [9.2, 12.0]	1.2 [1.0, 1.3]	1.5 [1.3, 1.7]	3.6 [2.6, 5.0]
		Women	16.8 [14.5, 19.5]	1.9 [1.6, 2.2]	2.3 [2.0, 2.7]	-
		Men	4.6 [3.5, 6.1]	0.5 [0.4, 0.7]	0.7 [0.5, 0.9]	-
	55–64	Both	10.7 [9.3, 12.5]	0.9 [0.7, 1.0]	1.3 [1.2, 1.6]	2.5 [1.8, 3.5]
		Women	15.4 [12.9, 18.3]	1.2 [1.0, 1.5]	1.9 [1.6, 2.3]	-
		Men	6.1 [4.7, 8.1]	0.5 [0.4, 0.7]	0.8 [0.6, 1.0]	-
	>64	Both	7.5 [6.5, 8.7]	0.6 [0.5, 0.7]	1.5 [1.3, 1.8]	2.4 [1.7, 3.4]
		Women	9.9 [8.4, 11.8]	0.9 [0.7, 1.0]	2.1 [1.8, 2.5]	-
		Men	4.5 [3.4, 5.9]	0.3 [0.3, 0.5]	0.9 [0.7, 1.2]	-
Histology type	Papillary	Both	5.7 [5.3, 6.1]	4.5 [4.2, 4.9]	5.5 [5.1, 5.9]	3.9 [3.3, 4.7]
		Women	9.1 [8.5, 9.9]	7.5 [6.9, 8.1]	8.9 [8.2, 9.6]	-
		Men	2.3 [1.9, 2.6]	1.7 [1.4, 2.0]	2.3 [1.9, 2.6]	-
	Follicular	Both	0.7 [0.6, 0.8]	0.5 [0.4, 0.7]	0.7 [0.6, 0.8]	4.2 [2.5, 7.1]
		Women	1.1 [0.9, 1.4]	0.9 [0.7, 1.1]	1.1 [0.9, 1.4]	-
		Men	0.3 [0.2, 0.4]	0.2 [0.1, 0.4]	0.3 [0.2, 0.4]	-
	Medullary	Both	0.4 [0.3, 0.5]	0.3 [0.2, 0.3]	0.4 [0.3, 0.5]	1.3 [0.7, 2.3]
		Women	0.4 [0.3, 0.6]	0.3 [0.2, 0.5]	0.4 [0.3, 0.6]	-
		Men	0.3 [0.2, 0.5]	0.2 [0.1, 0.3]	0.3 [0.2, 0.5]	-
	Anaplastic or poorly differentiated	Both	0.2 [0.1, 0.3]	0.1 [0.1, 0.2]	0.2 [0.1, 0.3]	0.9 [0.4, 1.9]
		Women	0.2 [0.1, 0.3]	0.1 [0.0, 0.2]	0.2 [0.1, 0.3]	-
		Men	0.2 [0.1, 0.3]	0.1 [0.1, 0.2]	0.2 [0.1, 0.4]	-
	Other	Both	0.1 [0.0, 0.1]	0.0 [0.0, 0.1]	0.1 [0.0, 0.1]	0.6 [0.2, 2.1]
		Women	0.1 [0.0, 0.2]	0.0 [0.0, 0.1]	0.1 [0.0, 0.2]	-
		Men	0.2 [0.0, 0.2]	0.1 [0.0, 0.1]	0.1 [0.0, 0.2]	-
Stage at diagnosis	I	Both	4.9 [4.6, 5.3]	4.1 [3.8, 4.5]	4.6 [4.3, 5.0]	4.8 [4.0, 5.9]
		Women	8.2 [7.5, 8.9]	7.0 [6.4, 7.6]	7.8 [7.2, 8.4]	-
		Men	1.7 [1.4, 2.0]	1.4 [1.1, 1.7]	1.6 [1.3, 1.9]	-
	II	Both	0.5 [0.4, 0.7]	0.4 [0.3, 0.5]	0.6 [0.5, 0.7]	3.0 [1.8, 5.0]
		Women	0.8 [0.6, 1.1]	0.6 [0.4, 0.7]	0.9 [0.7, 1.1]	-
		Men	0.3 [0.2, 0.4]	0.2 [0.1, 0.3]	0.3 [0.2, 0.5]	-
	III	Both	0.5 [0.4, 0.7]	0.4 [0.3, 0.4]	0.6 [0.5, 0.7]	2.6 [1.6, 4.3]
		Women	0.8 [0.6, 1.0]	0.5 [0.4, 0.7]	0.8 [0.6, 1.1]	-
		Men	0.3 [0.2, 0.5]	0.2 [0.1, 0.3]	0.3 [0.2, 0.5]	-
	IV	Both	0.7 [0.6, 0.8]	0.4 [0.3, 0.5]	0.7 [0.6, 0.9]	1.2 [0.8, 1.7]
		Women	0.8 [0.6, 1.0]	0.5 [0.3, 0.6]	0.8 [0.6, 1.0]	-
		Men	0.6 [0.5, 0.8]	0.4 [0.3, 0.5]	0.7 [0.5, 0.9]	-
	Unstaged	Both	0.3 [0.2, 0.4]	0.2 [0.1, 0.3]	0.3 [0.2, 0.4]	1.6 [0.8, 3.0]
		Women	0.4 [0.3, 0.6]	0.3 [0.2, 0.4]	0.4 [0.3, 0.5]	-
		Men	0.2 [0.1, 0.4]	0.1 [0.1, 0.2]	0.2 [0.1, 0.4]	-

* Incidence rates were expressed as cases per 100,000 person-years. ASRe: Age-standardized rate using the 2013 European population; ASRw: Age-standardized rate using the 1960 World population; CI: Confidence interval; CR: Crude rate; SR: Sex ratio.

## Data Availability

The original contributions presented in this study are included in the article/Supplementary Materials. Further inquiries can be directed to the corresponding author.

## Supplementary Materials

The following supporting information can be downloaded at https://www.mdpi.com/article/10.3390/cancers18193146/s1, Table S1: Annual population by sex for the province of Girona, 2002–2021. Table S2: Overdispersion, zero-inflation and goodness-of-fit test for Poisson assumption results for all Poisson generalized linear models (GLMs).

## Abbreviations

The following abbreviations are used in this manuscript:

TC	Thyroid cancer
CRs	Crude rates
ASRe	Age-standardized rate using the 2013 European population
ASRw	Age-standardized rate using the 1960 World population
APC	Annual percentage change
CIs	confidence intervals
GCR	Girona Cancer Registry
REDECAN	Spanish Network of Cancer Registries
ENCR	European Network of Cancer Registries
IACR	International Association for Cancer Registries
IARC	International Agency for Research on Cancer
ICD-O-3	International Classification of Diseases for Oncology, 3rd Edition
AJCC	American Joint Committee on Cancer
IQR	Interquartile range
ASRs	Age-standardized rates
SR	Sex ratio
GLM	Generalized linear model
ATA	American Thyroid Association
ER	Estrogen receptor
CCND1	cyclin D1
GPER1	G protein-coupled estrogen receptor 1
TSH	Thyroid-stimulating hormone
hCG	Human chorionic gonadotropin
EDC	Endocrine-disrupting chemical
PFAS	Per-/poly-fluoroalkyl substances
PCBs	Polychlorinated biphenyls
