# Supplementary material for: Thyroid Cancer Incidence in North-East Spain, 2002–2021: A Population-Based Study
Source: Cancers (Basel). 2026 Sep 29;18(19):3146. doi: 10.3390/cancers18193146 (PMC13664602; doi:10.3390/cancers18193146)
Supplement: Supplementary file 1 [file cancers-18-03146-s001.zip › cancers-4472948-supplementary.pdf]

Table S1: Annual population by sex for the province of Girona, 2002–2021.

Year	Men	Women
2002	289,615	291,139
2003	303,287	302,419
2004	316,779	313,541
2005	332,806	325,545
2006	344,925	336,390
2007	359,322	348,246
2008	370,875	358,281
2009	375,334	364,034
2010	376,598	367,669
2011	378,470	371,026
2012	377,577	372,736
2013	374,396	371,683
2014	370,785	370,286
2015	369,961	370,463
2016	370,421	372,137
2017	372,493	374,869
2018	374,559	377,950
2019	379,222	382,260
2020	383,344	386,327
2021	385,420	388,367

Table S2: Overdispersion, zero-inflation and goodness-of-fit test for Poisson assumption results for all Poisson generalized linear models (GLM).

**Poisson GLM**	**Overdispersion Ratio, χ^2, *p*-value**	**Zero-inflation Predicted zero/Observed zero**	**Goodness-of-fit test for Poisson assumption χ^2, df, *p*-value**
Overall	0.88, $\chi^2 = 300.00$, $p = 0.95$	103/100 (Ratio = 1.03)	$\chi^2 = 310.49$, df = 341, $p = 0.89$
Sex			
Women	0.93, $\chi^2 = 316.13$, $p = 0.83$	120/122 (Ratio = 0.98)	$\chi^2 = 322.97$, df = 341, $p = 0.75$
Men	0.84, $\chi^2 = 286.40$, $p = 0.99$	221/215 (Ratio = 1.03)	$\chi^2 = 268.39$, df = 341, $p = 0.99$
Age group (years)			
<35	0.70, $\chi^2 = 92.55$, $p = 0.99$	73/70 (Ratio = 1.04)	$\chi^2 = 89.23$, df = 132, $p = 0.99$

35 – 44	0.91, $\chi^2 = 33.64$, p = 0.63	No observed zeros	$\chi^2 = 34.46$, df = 37, p = 0.59
45 – 54	0.98, $\chi^2 = 36.12$, p = 0.51	No observed zeros	$\chi^2 = 37.66$, df = 37, p = 0.44
55 – 64	0.94, $\chi^2 = 34.68$, p = 0.58	1/1 (Ratio = 1.00)	$\chi^2 = 38.55$, df = 37, p = 0.40
>64	1.12, $\chi^2 = 105.45$, p = 0.20	29/29 (Ratio = 1.00)	$\chi^2 = 104.76$, df = 94, p = 0.21
Histology			
Papillary	0.88, $\chi^2 = 301.32$, p = 0.94	123/123 (Ratio = 1.00)	$\chi^2 = 312.00$, df = 341, p = 0.87
Follicular	0.87, $\chi^2 = 294.80$, p = 0.97	281/278 (Ratio = 1.01)	$\chi^2 = 229.24$, df = 341, p = 0.99
Medullary	0.76, $\chi^2 = 260.34$, p = 0.99	316/316 (Ratio = 1.00)	$\chi^2 = 167.19$, df = 341, p = 0.99
Anaplastic	0.58, $\chi^2 = 199.31$, p = 0.99	337/335 (Ratio = 1.01)	$\chi^2 = 89.79$, df = 341, p = 0.99
Other	0.45, $\chi^2 = 153.64$, p = 0.99	349/349 (Ratio = 1.00)	$\chi^2 = 55.66$, df = 341, p = 0.99
Stage at diagnosis			
I	0.80, $\chi^2 = 270.97$, p = 0.99	143/144 (Ratio = 0.99)	$\chi^2 = 286.60$, df = 341, p = 0.99
II	0.73, $\chi^2 = 249.24$, p = 0.99	302/307 (Ratio = 0.98)	$\chi^2 = 171.11$, df = 341, p = 0.99
III	0.66, $\chi^2 = 225.27$, p = 0.99	302/305 (Ratio = 0.99)	$\chi^2 = 174.45$, df = 341, p = 0.99
IV	0.71, $\chi^2 = 241.30$, p = 0.99	284/281 (Ratio = 1.01)	$\chi^2 = 201.10$, df = 341, p = 0.99
Unstaged	0.88, $\chi^2 = 299.78$, p = 0.95	322/320 (Ratio = 1.01)	$\chi^2 = 151.99$, df = 341, p = 0.99
